# Prospective Italian real‐world study of mepolizumab in severe eosinophilic asthma validates retrospective outcome reports

**DOI:** 10.1002/clt2.12067

**Published:** 2021-10-14

**Authors:** Laura Pini, Cristiano Caruso, Stefania Colantuono, Diego Bagnasco, Aoife Maxwell, Robert G. Price, Peter Howarth, Giorgio Walter Canonica, Italian investigators of REALITI‐A

**Affiliations:** ^1^ Respiratory Unit Spedali Civili di Brescia Brescia Italy; ^2^ Department of Clinical and Experimental Sciences University of Brescia Brescia Italy; ^3^ Department of Medical and Surgical Sciences Fondazione Policlinico A. Gemelli IRCCS Rome Italy; ^4^ Università Cattolica del Sacro Cuore Rome Italy; ^5^ Allergy and Respiratory Diseases IRCCS Policlinico San Martino University of Genoa Genoa Italy; ^6^ Real World Study Delivery Value Evidence and Outcomes Global Medical GSK Stevenage Hertfordshire UK; ^7^ Biostatistics R&D GSK Stevenage Hertfordshire UK; ^8^ Global Medical Global Specialty & Primary Care TA GSK House Brentford Middlesex UK; ^9^ Department of Biomedical Sciences Humanitas University Milan Italy; ^10^ Personalized Medicine Asthma and Allergy Clinic Humanitas Research Hospital IRCCS Milan Italy

To the Editor,

1

Current asthma guidelines inform about evidence‐based approaches to optimize patient outcomes. These are primarily based on randomized controlled trials (RCTs) conducted within well‐defined but restricted populations.^1^ In real life, healthcare professionals encounter complex clinical, lifestyle, psychosocial, demographic and attitudinal factors in patient care; aspects of the real world that are not accommodated within RCTs. The therapeutic outcomes observed in such real‐world everyday clinical settings will reflect the integrated interaction of a medicine's proven efficacy with factors related to patients' actual medication use, and the healthcare system in which the treatment is used. As such, real‐world experience (RWE) data is critical to extrapolating the results of RCTs to multifaceted patient populations, confirming or refuting effectiveness.^2^

Real‐life studies of the anti‐interleukin‐5 biologic, mepolizumab, are an important part of healthcare utilization evaluation and a number of RWE studies have been reported in severe eosinophilic asthma (SEA), including those from France, Australia and the ongoing global ‘REALITI‐A’ study, to which centres in Italy contributed.^3^, ^4^, ^5^

REALITI‐A is a prospective study, whereas many other RWE studies gather data retrospectively. As Italy was a significant contributor to REALITI‐A and the Severe Asthma Network in Italy (SANI) has published retrospectively assessed data of RWE mepolizumab outcomes,^6^ there is an opportunity to compare outcomes with mepolizumab in SEA as evaluated both prospectively and retrospectively in the same healthcare system. Here we report outcome data from the Italian participants in the early initiator interim analysis of the REALITI‐A study and compare them with distinct patients from the SANI retrospective outcome report.

REALITI‐A is an ongoing, 2‐year, global, prospective, single‐arm, observational cohort study assessing the effectiveness of mepolizumab (100 mg subcutaneously) as used in a real‐world setting for patients with SEA.^5^ Eighty‐seven (23.6%) of the 368 participants in the REALITI‐A study, reported so far, were recruited from 14 Italian centres. Baseline demographics from the overall REALITI‐A study population and from participants in the SANI retrospective analysis of mepolizumab treatment outcomes, are shown in Table 1.

**TABLE 1:** Demographics and characteristics of patients

Parameter	REALITI‐A^5^	REALITI‐A	SANI study^6^
	Total population	Italian cohort	Italian population
Number of subjects	368	87	106
Age (years), mean (SD)	53 (13.7)	56 (10.8)	57 (10.6)
Female, *n* (%)	226 (62)	54 (62)	60 (57)
Never smokers, *n* (%)	221 (61)	55 (63)	89 (84)
Exacerbations in the previous year, mean (SD)	4.7 (4.1)	3.5 (2.5)	4.1 (2.7)
Receiving maintenance OCS at enrolment, *n* (%)	174 (48)	39 (45)	84 (79)
Prednisone equivalent OCS dose, (mg/day), mean (SD)	14.4 (19.5)	9.9 (7.6)	9.5 (8.5)
Comorbid nasal polyps, *n* (%)	140 (38)	54 (62)	65 (61)
Blood eosinophils (cells/μl), geometric mean	370	572	674
Previous treatment with Omalizumab, *n* (%)	71 (19)	15 (17)	Data not available
Withdrew from treatment during 12‐month follow‐up period, *n* (%)	70 (19)	2 (2)	0
Withdrew from treatment due to lack of efficacy, during 12‐month follow‐up period, *n* (%)	13 (4)	0	0

Abbreviations: OCS, oral corticosteroid; SANI, Severe Asthma Network in Italy; SD, standard deviation.

In the Italian participants of the REALITI‐A study, 12 months of treatment with mepolizumab was associated with clinical improvement (Figure 1). There was an 80% reduction in severe exacerbations (from mean of 3.48/year to 0.69/year, rate ratio [RR] 0.20 [95% confidence interval {CI} 0.14–0.27]) and a reduction in emergency room/hospitalization events of 84% (from 0.44/year to 0.07/year, RR 0.16 [95%CI 0.04–0.58]). Mepolizumab reduced the median maintenance prednisone equivalent oral corticosteroid (OCS) use from 10 mg/day at baseline to 2.5 mg/day between Weeks 45 and 56. These results are comparable to the RWE outcomes with mepolizumab therapy in 106 Italian patients with SEA reported by the SANI.^6^ In this retrospective report, the introduction of mepolizumab therapy was associated with an 81% decrease in severe exacerbations (from 4.09/year to 0.77/year, RR 0.19 [95%CI 0.15–0.24]) and an 85.4% decrease in hospitalization for severe asthma (from 0.39/year to 0.06/year, RR 0.15 [95%CI 0.05–0.35]. Furthermore, 60.7% of those on OCS at baseline (*n* = 84) were able to completely discontinue this treatment over 1 year. No significant adverse events were described during this observation period and no Italian patient discontinued mepolizumab due to an adverse event in either the SANI or REALITI‐A studies.

**FIGURE 1:**
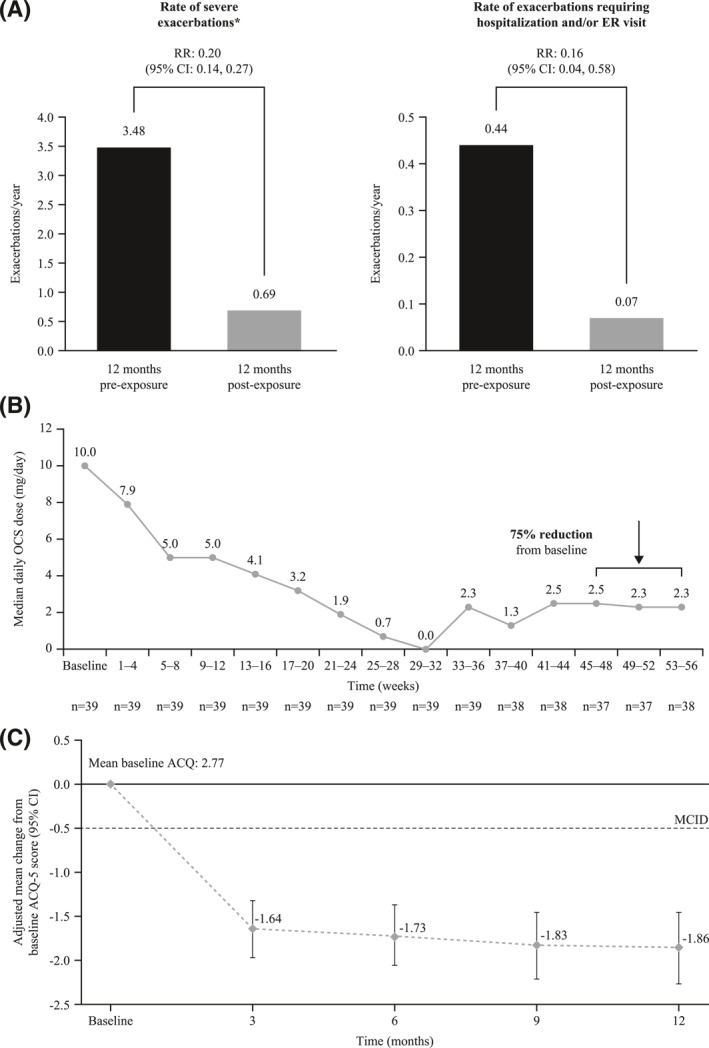
Outcomes from the Italian participants in the early initiator analysis of the REALITI‐A study: (A) exacerbation rates, (B) OCS dose, (C) ACQ‐5 score. *Defined as a deterioration in asthma requiring SCS (any dose; oral steroids, e.g. prednisone) for ≥3 days or a single systemic administration of corticosteroids (intravenous/intramuscular dose) and/or hospitalization and/or ER visit. For participants on maintenance systemic corticosteroids (without a hospitalization and/or ER visit), at least double the existing maintenance dose for at least 3 days is required. Analysis of exacerbation rate performed using generalized estimating equation model assuming a negative binomial distribution, with covariate of treatment period (pre‐treatment, on‐treatment). The logarithm of time is used as an offset variable. Analysis of ACQ‐5 score performed using mixed model repeated measures with covariates of time point, baseline OCS therapy (use, no use) and number of exacerbations pre‐exposure (0, 1, 2, 3, ≥4). ACQ, Asthma Control Questionnaire; CI, confidence interval; ER, emergency room; MCID, minimal clinically important difference; OCS, oral corticosteroid; RR, rate ratio; SCS, systemic corticosteroid

It should be noted, however, that there are some disparities between the two studies, such as differences in OCS use (45% vs. 79%) and geometric mean blood eosinophil counts (572 cells/μl vs. 674 cells/μl). However despite these differences both studies were RWE studies, patients were selected by physicians, and both studies enrolled patients over similar periods of time (December 2016–February 2019 [REALITI‐A] vs. May 2017–December 2018 [SANI]). Notably, the severity of asthma experienced by patients in both studies was very similar, and additionally patients had similar annual exacerbation rates (3.48 vs. 4.09 exacerbations/year) and similar hospitalization rates (0.44/year vs. 0.39/year).

The Italian prospective REALITI‐A findings strongly support the SANI retrospective study outcomes and indicate that retrospective data gathering did not lead to an overemphasis of impact. These findings showcase the value of severe asthma networks to gather RWE in populations treated in standard clinical care. Furthermore, the findings have healthcare resource and cost implications, due to the positive impact of mepolizumab in reducing severe exacerbations and oral steroid dependency.

## CONFLICTS OF INTEREST

Laura Pini reports having received research grants and lecture fees from A. Menarini, AstraZeneca, Chiesi Farmaceutici, GSK, and Novartis; Cristiano Caruso reports having received research grants and lecture fees from GSK and AstraZeneca; Stefania Colantuono reports no conflicts of interests; Diego Bagnasco reports having received lecture fees from, AstraZeneca, GSK, Novartis, and Sanofi‐Genzyme; Peter Howarth, Aoife Maxwell, and Robert G. Price are employees of GSK and own stocks/shares in GSK; Giorgio Walter Canonica reports research grants and fees from: A. Menarini, Alk‐Abello’, Allergy Therapeutics, AstraZeneca, Boehringer Ingelheim, Chiesi Farmaceutici, Genentech, Guidotti‐Malesci, GSK, Hal Allergy, Mylan, Merck, Merck Sharp & Dome, Mundipharma, Novartis, Regeneron, Roche, Sanofi‐Aventis, Sanofi‐Genzyme, Stallergenes‐Greer, UCB Pharma, Uriach Pharma, Valeas, and Vibor‐Pharma.
